# Efficacy of ribavirin against malignant glioma cell lines

**DOI:** 10.3892/ol.2014.2569

**Published:** 2014-09-26

**Authors:** AKIYOSHI OGINO, EMIKO SANO, YUSHI OCHIAI, SHUN YAMAMURO, SHINYA TASHIRO, KAZUNARI YACHI, TAKASHI OHTA, TAKAO FUKUSHIMA, YUTAKA OKAMOTO, KOUHEI TSUMOTO, TAKUYA UEDA, ATSUO YOSHINO, YOICHI KATAYAMA

**Affiliations:** 1 Department of Neurological Surgery, Nihon University School of Medicine, Tokyo 173-8610, Japan; 2 Department of Medical Genome Sciences, Graduate School of Frontier Sciences, The University of Tokyo, Chiba 277-8562, Japan; 3 Medical Proteomics Laboratory, The Institute of Medical Science, The University of Tokyo, Tokyo 108-8639, Japan; 4 New Energy and Industrial Technology Development Organization, Kawasaki 212-8554, Japan

**Keywords:** ribavirin, malignant glioma

## Abstract

Ribavirin (1-β-D-ribofuranosy-1,2,4-triazole-3-carboxamide) has been widely administered as an antiviral agent against RNA and DNA viruses. Ribavirin, in combination with interferon, has predominantly been applied in the treatment of the hepatitis C virus infection and its potential antitumor efficacy has recently become a point of interest. The aim of the present study was to evaluate the effect of ribavirin on the growth of malignant glioma cells, to identify novel predictive genes in malignant glioma cells (by analyzing gene expression profiles) and to assess the influence of ribavirin on the cell cycle of malignant glioma cells. The present study evaluated the antitumor efficacy of ribavirin against various malignant glioma cell lines (A-172, AM-38, T98G, U-87MG, U-138MG, U-251MG and YH-13). After culturing the cells in ribavirin-containing culture medium (final concentration, 0–1,000 μM) for 72 h, the viable proliferated cells were harvested and counted. The half maximal inhibitory concentration of ribavirin, with regard to the growth of the malignant glioma cell lines, was determined from the concentration of ribavirin required for 50% growth inhibition in comparison to the untreated control cells. Furthermore, the current study identified the genes in which the gene expression levels correlated with the ribavirin sensitivity of the malignant glioma cells lines, using a high-density oligonucleotide array. Finally, cell cycle analysis was performed on the U-87MG cell line. It was identified that ribavirin inhibited the growth of all of the malignant glioma cell lines in a dose-dependent manner, although the ribavirin sensitivity varied between each cell line. Of the extracted genes, *PDGFRA* demonstrated the strongest positive correlation between gene expression level and ribavirin sensitivity. Cell cycle analysis of the U-87MG cell line demonstrated that ribavirin treatment induces G0/G1 arrest and thus may be an effective agent for inhibiting malignant glioma cell growth. Therefore, the results of the current study indicate that ribavirin may have potential as a therapeutic agent in the treatment of malignant gliomas.

## Introduction

Ribavirin (1-β-D-ribofuranosy-1,2,4-triazole-3-carboxamide) was developed as an antiviral agent against RNA and DNA viruses and its function was first described in 1972 by Sidwell *et al* (1). Ribavirin, in combination with interferon (IFN)*,* is one of the standard treatment strategies for hepatitis C virus (HCV) infections (2). Ribavirin was expected to be clinically advantageous in the treatment of various viral infections, however, to date the clinical utilization of ribavirin has been limited to the treatment of HCV infections.

Recently, interest in the efficacy of ribavirin for the treatment of tumors has increased due to two reasons. Firstly, ribavirin inhibits inosine-5′-monophosphate dehydrogenase (IMPDH) (3). IMPDH is a key enzyme in guanosine triphosphate synthesis, and IMPDH expression levels and activity are elevated in certain types of tumor. Furthermore, IMPDH is also associated with the proliferation and transformation of malignant tumor cells (4). Therefore, an IMPDH inhibitor may be a candidate for antitumor chemotherapy. Secondly, ribavirin inhibits the eukaryotic translation initiation factor 4E (eIF4E) (5,6). eIF4E has two major functions in gene expression: Messenger (m)RNA translation and mRNA export (7). The eIF4E protein is present in the nucleus and the cytoplasm; in the nucleus, eIF4E facilitates the export of a subset of specific growth-promoting mRNAs. In the cytoplasm, eIF4E recruits mRNA with highly structured 5′-untranslated regions to the ribosome to promote translation. Therefore, eIF4E, which is overexpressed in ~30% of human cancers (5,8), may have oncogenic potential.

The antitumor efficacy of ribavirin has been reported in breast cancer and leukemia (5,9), however, to the best of our knowledge it has yet to be reported in malignant glioma. The aim of the present investigation was to evaluate the antitumor efficacy of ribavirin on malignant glioma cells, to identify novel predictive genes for ribavirin sensitivity through the evaluation of gene expression profiles and to assess the influence of ribavirin on the cell cycle of malignant glioma cells.

## Materials and methods

### Cell lines and cell culture

Human malignant glioma cells of the A-172, AM-38, T98G, U-251MG and YH-13 cell lines were obtained from Health Science Research Resources Bank (Osaka, Japan), and the U-87MG and U-138MG cell lines were purchased from the American Type Culture Collection (Manassas, VA, USA). All of the cell lines were cultured in Dulbecco’s modified Eagle’s medium (Nissui Pharmaceutical, Tokyo, Japan) supplemented with 10% fetal bovine serum (Life Technologies, Grand Island, NY, USA) in a standard humidified incubator at 37°C with an atmosphere of 5% CO_2_.

### Cell culture growth with ribavirin

Malignant glioma cell proliferation was evaluated using a Z1 Coulter Counter^®^ (Beckman Coulter, Brea, CA, USA) to count the cell growth in 24-well plates (Iwaki, Chiba, Japan). Each well was seeded with 1×10^4^ cells and cultured for 24 h prior to ribavirin treatment to allow adherence of the cells to the plate. The culture medium was replenished with fresh medium containing ribavirin (0.1–1,000 μM), and the cells were cultured for 72 h. The proliferated cells were trypsinized with trypsin-EDTA solution (Invitrogen Life Technologies, San Diego, CA, USA) and counted using the Z1 Coulter Counter^®^. The cell culture growth experiments were repeated a minimum of seven times at each concentration. The half maximal inhibitory concentration (IC_50_), with regard to the growth of the malignant glioma cell culture, was determined from the concentration of ribavirin required for 50% growth inhibition in comparison to untreated control cells.

### RNA preparation and hybridization

Total RNA was extracted from the malignant glioma cells using an RNeasy^®^ mini kit (Qiagen, Valencia, CA, USA) and quantified by spectrophotometry (Jasco V-550 spectrophotometer; Jasco Interntational Co., Ltd., Tokyo, Japan). Double-stranded complementary (c)DNA was generated from the total RNA (5 μg) using a One-Cycle cDNA Synthesis kit (Affymetrix, Inc., Santa Clara, CA, USA). Biotinylated cRNA was then synthesized from the cDNA in an *in vitro* transcription reaction (IVT) by employing an IVT labeling kit (Affymetrix, Inc.). The biotinylated cRNA (10 μg) was fragmented and hybridized to a DNA oligonucleotide expression array (Human Genome U133A 2.0 Array; Affymetrix, Inc.) containing >22,277 probe sets for ~14,500 human genes (certain genes are represented on the array by multiple probe sets). The hybridized probe array was washed and stained with a streptavidin-phycoerythrin conjugate (Molecular Probes Life Technologies, Carlsbad, CA, USA) using a GeneChip^®^ Fluidics Station 450 (Affymetrix, Inc.) according to the manufacturer’s instructions, as previously described (10,11).

### Identification of discriminatory genes for ribavirin sensitivity

The probe array was scanned with a confocal laser scanner (GeneChip^®^ Scanner 3000; Affymetrix, Inc.) and analyzed using GeneChip^®^ Operating Software 1.1 (Affymetrix, Inc.), allowing the gene expression levels to be calculated from the signal intensities. All of the genes represented on the GeneChip^®^ were globally normalized and scaled to the signal intensity to provide a gene expression value. To avoid contributions from artificial sources of variation in the experimentally measured expression patterns, each cell line was grown in three independent cultures and the entire process was conducted independently on mRNA that was extracted from each culture. The expression array analysis for each cell line was then run in triplicate.

### Cell cycle distribution analysis

The cells were plated at 2×10^5^ cells per 25-cm^2^ flask (Iwaki) and incubated for 24 h to allow attachment of the cells to the flask. The culture medium was then replenished with fresh medium containing 10 μM ribavirin and the cells were harvested using trypsin-EDTA solution at 0, 4, 8 and 24 h. The cells were rinsed with phosphate-buffered saline, fixed using ice-cold 70% ethanol, incubated with 200 μg/ml RNase A (Roche Diagnostics, Basel, Switzerland) for 30 min at room temperature and stained with 2 μg/ml propidium iodide (PI) solution (Miltenyi Biotec, Inc., San Diego, CA, USA). The fluorescence was measured by flow cytometry, using the BD FACSCalibur™ flow cytometer (BD Biosciences, Franklin Lakes, NJ, USA), at a fluorescence wavelength of 610 nm. The resulting DNA histogram was analyzed using FlowJo software (BioLegend, Inc., San Diego, CA, USA).

### Statistical analysis

To identify the discriminative genes for ribavirin sensitivity, the Pearson’s correlation test was performed to evaluate the association between the IC_50_ of ribavirin and the gene expression level of each gene (Microsoft Office Excel 2007, Redmond, WA, USA).

## Results

### Antitumor efficacy of ribavirin

**Figure 1:**
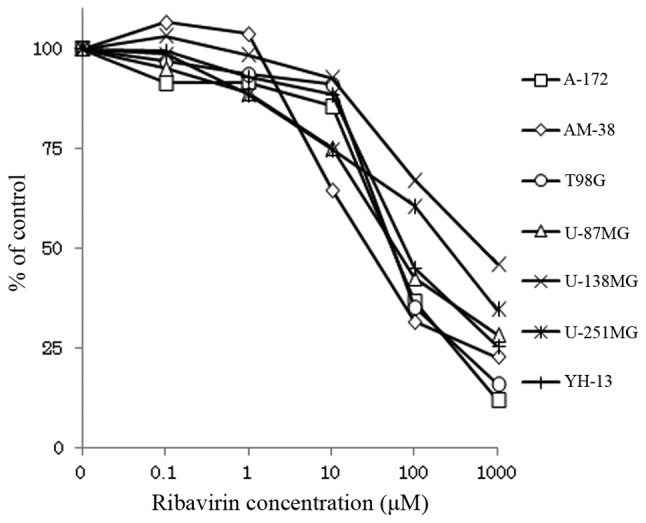
Antitumor effect of ribavirin on seven malignant glioma cell lines. At 72 h, following the addition of ribavirin (final concentration, 0–1,000 μM) to the culture medium, the number of viable cells was counted and expressed as a percentage of the untreated control cells.

Seven malignant glioma cell lines were treated with 0.1–1,000 μM ribavirin and cultured for 72 h. The proliferated cells were trypsinized and counted using a Z1 Coulter Counter^®^. As demonstrated by Fig. 1, the growth of all of the cell lines was inhibited by ribavirin in a dose-dependent manner, although the sensitivity of each cell line to ribavirin varied. The IC_50_ of ribavirin for five of the malignant glioma cell lines (A-172, AM-38, T98G, U-87MG and YH-13) was <100 μM, whereas, the IC_50_ for the other two cell lines (U-138MG and U-251MG) was >250 μM (Table I).

### Identification of discriminatory genes for ribavirin sensitivity

**Figure 2:**
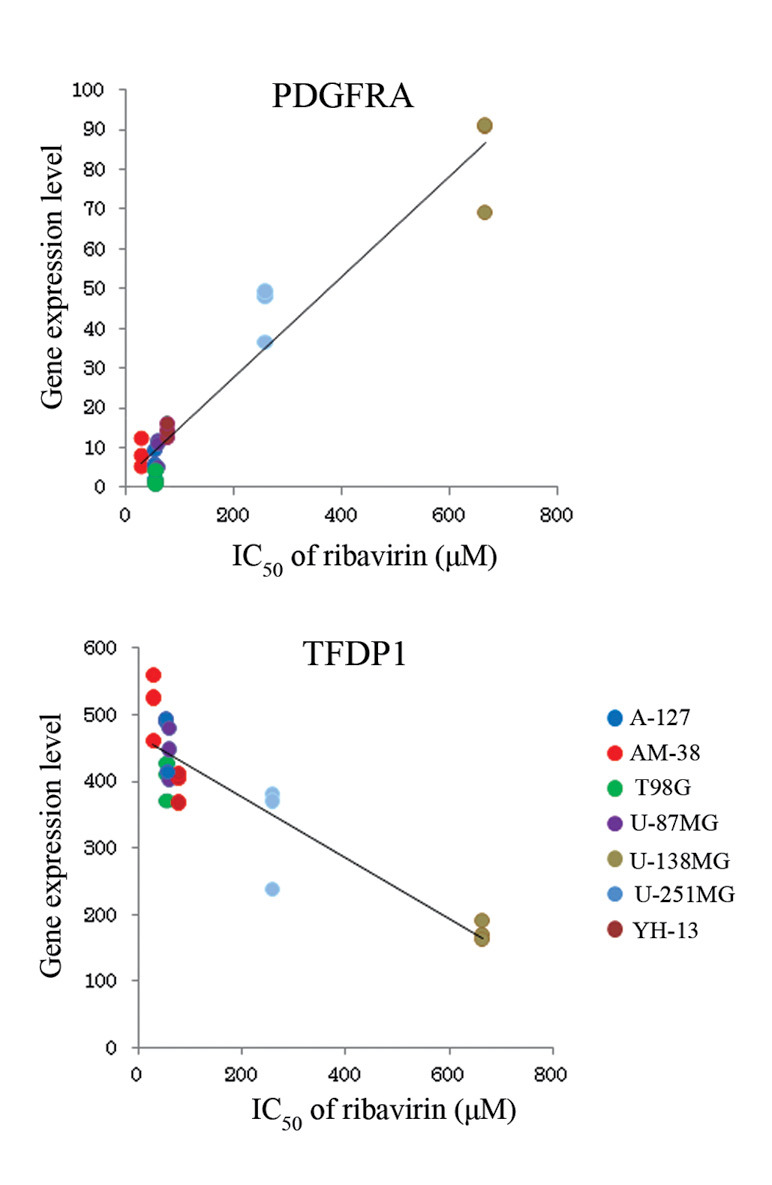
Association between gene expression levels (*PDGFRA* and *TFDP1*) and ribavirin sensitivity (IC_50_ of ribavirin) of seven malignant glioma cell lines. IC_50_, half maximal inhibitory concentration.

Of the 22,277 probe sets, genes that were not expressed were omitted, therefore, 16,913 probe sets were available for subsequent analysis. The expression profiling data for the malignant glioma cell lines were consistent with data from previous reports (10,11). Various genes that were expressed in the seven malignant glioma cell lines included in the present study were observed to be upregulated or downregulated relative to ribavirin sensitivity. The negative and positive correlation values of the association between gene expression level and ribavirin sensitivity are indicated in Table II by the Pearson’s correlation coefficient and the corresponding P-value. The gene expression levels of *PDGFRA* and *TFDP1* indicate the greatest positive and negative correlation with ribavirin sensitivity, respectively (Fig. 2).

### Cell cycle distribution analysis

**Figure 3:**
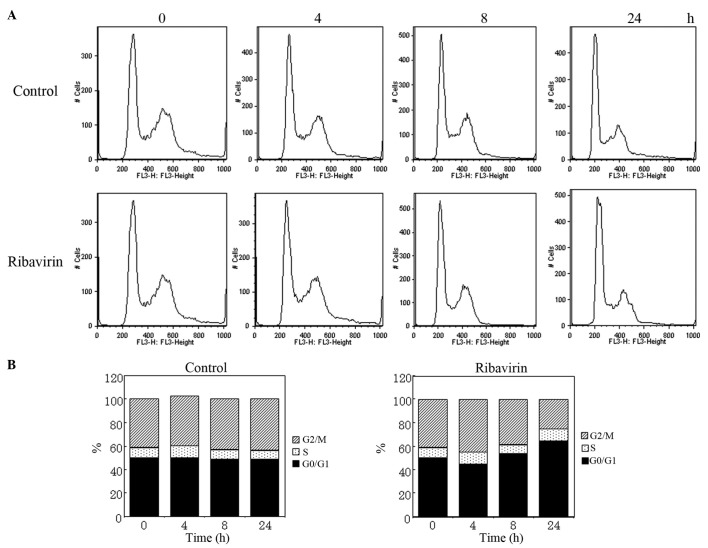
Cell cycle distribution analysis of U-87MG cells, which were treated with ribavirin (with untreated cells serving as the control). (A) Fluorescence-activated cell sorting plot and (B) histograms indicating the antitumor effects.

To clarify the antitumor efficacy of ribavirin on malignant glioma cells, alterations in the cell cycle distribution were examined using U-87MG cells treated with ribavirin. Unsynchronized cells were treated with 10 μM ribavirin for 4, 8 and 24 h. The harvested cells were fixed using ethanol, treated with RNase A and stained with PI for analysis by fluorescence-activated cell sorting (FACS; Fig. 3A). Histograms presenting the FACS data (Fig. 3B) demonstrate an increase in the population of cells in the G0/G1 phase following treatment with ribavirin, with time-lapse indicating that the antitumor efficacy of ribavirin results from the accumulation of cells in the G0/G1 phase.

## Discussion

To the best our knowledge, the current study is the first to demonstrate that ribavirin inhibits the growth of malignant glioma cells. Ribavirin was initially developed as an antiviral agent against RNA and DNA viruses and is predominantly used, in combination with IFN, as a treatment strategy for HCV infection (2). Although ribavirin was expected to be developed as a therapeutic treatment for various other viral infections, its clinical use has thus far been restricted to the treatment of HCV infections. Interest in the antitumor efficacy of ribavirin has been increasing due to its ability to inhibit IMPDH and eIF4E (1,6). Furthermore, the antitumor efficacy of ribavirin has been reported in the treatment of breast cancer and leukemia (5,9).

The antitumor efficacy of ribavirin on malignant glioma cell lines was measured and it was revealed that the growth of these cell lines was inhibited by ribavirin in a dose-dependent manner. Of the seven malignant glioma cell lines investigated in the current study, the IC_50_ of five of these cell lines (A-172, AM-38, T98G, U-87MG and YH-13) was <100 μM and the IC_50_ of the other two glioma cell lines (U-138MG and U-251MG) was >250 μM. Based on these data, the malignant glioma cell lines can be divided into ribavirin-effective and ribavirin-resistant groups. The genes that were positively and negatively correlated with ribavirin sensitivity are listed in Table II. To the best of our knowledge, none of the genes identified in the current study were previously expected to be associated with ribavirin or chemotherapy sensitivity. *TFDP1* has been associated with the cell cycle and seven genes (*PDGFRA*, *ZEP36L2*, *STS*, *TFDP1*, *HAS2*, *FBL* and *KLF5*) have previously been associated with cell proliferation. In the present study, *PDGFRA* demonstrated the greatest positive correlation between gene expression level and the IC_50_ of ribavirin. Platelet-derived growth factor (PDGF) is a major mitogen for glial cells and connective tissue, and is key in the development of the central nervous system, wound healing, inflammation and neoplasia (12). PDGF receptor α is the cell surface receptor of PDGF. *PDGFRA* mRNA overexpression was detected in the low- and high-grade astrocytomas (13) and *PDGFRA* amplification is typical in the signaling pathway that results in the development of secondary glioblastoma (14). PDGFRA amplification is important for subgroup classification of malignant gliomas. To the best our knowledge, PDGFRA amplification and overexpression has not been reported, with regard to its association with chemotherapy sensitivity. However, although the association between rebavirin and PDGFRA amplification is unclear, we hypothesize that PDGFRA expression may be involved in the antitumor efficacy of ribavirin. Although it has been reported that ribavirin has an effect on the types of breast cancer that overexpress eIF4E, eIF4E was not extracted and analyzed in the present study as eIF4E is upregulated in all high-grade astrocytic tumors (15) and hence all malignant glioma cell lines. Furthermore, IMPDH was not included in the present study as it was reported that ribavirin inhibits leukemic cell proliferation through multiple signaling pathways (16), therefore, ribavirin may act on malignant glioma cells via mechanisms that do not involve eIF4E or IMPDH.

Finally, a cell cycle analysis of the harvested U-87MG cells, which were treated with ribavirin revealed that cells accumulated at the G0/G1 boundary; however, the sub-G1 population, which indicates apoptotic cells, did not increase. Vallée *et al* (17) reported that, in a melanoma cell line, the cell cycle was blocked in the G0/G1 phase by treatment with 100 μM ribavirin. The results of the present study also indicate that ribavirin is important in the inhibition of cell growth in malignant glioma by inducing G0/G1 arrest. To the best of our knowledge, no studies have reported that ribavirin alone is able to induce apoptosis in malignant glioma cells. However, Schlosser *et al* (18) reported that the number of human hepatoma apoptotic cells was increased by treatment with 50 μM ribavirin plus α-IFN. This synergistic effect is also expected to occur in malignant glioma cells treated with ribavirin combined with IFN or temozolomide (TMZ).

The present standard postoperative treatment strategy for malignant glioma is TMZ plus radiation and the median treatment survival time is 14.6 months (19). The results of the current study indicate that ribavirin may present as a novel agent for malignant glioma chemotherapy and that *PDGFRA* expression levels may be a significant marker of the antitumor efficacy of ribavirin against malignant gliomas.

## Figures and Tables

**Table I:** IC_50_ of ribavirin for malignant glioma cell lines.

Cell line	IC_50_, μM
A-172	53.6
AM-38	27.9
T98G	55.0
U-87MG	59.7
U-138MG	664.2
U-251MG	257.7
YH-13	76.9

IC_50_, half maximal inhibitory concentration.

**Table II:** Differentially expressed genes associated with ribavirin sensitivity.

A, Positive differentially expressed genes			

Symbol	Gene title	Correlation	P-value
*PDGFRA*	Platelet-derived growth factor receptor, α polypeptide 0.968	<0.0001	
*ZFP36L2*	Zinc finger protein 36, C3H type-like 2	0.951	<0.0001
*UBL3*	Ubiquitin-like 3	0.944	<0.0001
*HSPB2*	Heat shock 27 kDa protein 2	0.940	<0.0001
*HERC2P2*, *HERC2P3*	Hect domain and RLD 2 pseudogene 2; Hect domain and RLD 2 pseudogene 3	0.940	<0.0001
*MEOX2*	Mesenchyme homeobox 2	0.933	<0.0001
*SDC2*	Syndecan 2	0.929	<0.0001
*POSTN*	Periostin, osteoblast specific factor	0.921	<0.0001
*PLCB1*	Phospholipase C, β 1 (phosphoinositide-specific)	0.920	<0.0001
*KCNJ8*	Potassium inwardly-rectifying channel, subfamily J, member 8	0.918	<0.0001
*MAP4K4*	Mitogen-activated protein kinase kinase kinase kinase 4	0.917	<0.0001
*DOK5*	Docking protein 5	0.916	<0.0001
*STS*	Steroid sulfatase (microsomal), isozyme S	0.913	<0.0001
*HAS2*	Hyaluronan synthase 2	0.912	<0.0001
*AP1G1*	Adaptor-related protein complex 1, γ 1 subunit	0.911	<0.0001

B, Negative differentially expressed genes			

Symbol	Gene title	Correlation	P-value

*TFDP1*	Transcription factor Dp-1	−0.894	<0.0001
*OSBPL9*	Oxysterol binding protein-like 9	−0.806	<0.0001
*PLAA*	Phospholipase A2-activating protein	−0.796	<0.0001
*BTF3*	Basic transcription factor 3	−0.795	<0.0001
*PPWD1*	Peptidylprolyl isomerase domain and WD repeat containing 1	−0.792	<0.0001
*LARS*	Leucyl-tRNA synthetase	−0.792	<0.0001
*NDUFA8*	NADH dehydrogenase (ubiquinone) 1 α subcomplex, 8, 19 kDa	−0.790	<0.0001
*ERAP2*	Endoplasmic reticulum aminopeptidase 2	−0.787	<0.0001
*PTPN4*	Protein tyrosine phosphatase, non-receptor type 4 (megakaryocyte)	−0.784	<0.0001
*HMGCR*	3-hydroxy-3-methylglutaryl-CoA reductase	−0.781	<0.0001
*TAF9*	TAF9 RNA polymerase II, TBP-associated factor, 32 kDa	−0.777	<0.0001
*KLF5*	Kruppel-like factor 5 (intestinal)	−0.774	<0.0001
*XRCC4*	X-ray repair complementing defective repair in Chinese hamster cells 4	−0.769	<0.0001
*MSH3*	MutS homolog 3	−0.762	<0.0001
*FBL*	Fibrillarin	−0.753	<0.0001

tRNA, transfer RNA; NADH, nicotinamide adenine dinucleotide (reduced form); CoA, coenzyme A; TBP, TATA box-binding protein.
